# Chronic Inflammatory Demyelinating Polyneuropathy Post-mRNA-1273 Vaccination

**DOI:** 10.7759/cureus.24528

**Published:** 2022-04-27

**Authors:** Simranjit Singh, Fnu Sanna, Ramesh Adhikari, Ramya Akella, Karthik Gangu

**Affiliations:** 1 Medicine, Division of General Internal Medicine and Geriatrics, Indiana University School of Medicine, Indianapolis, USA; 2 Hospital Medicine, Franciscan Health, Lafayette, USA; 3 Geriatrics, Brown University, Providence, USA; 4 Internal Medicine, Pikeville Medical Center, Pikeville, USA; 5 Internal Medicine, University of Missouri, Columbia, USA; 6 Internal Medicine, University of Missouri Health Care, Columbia, USA

**Keywords:** mrna-1273, moderna mrna-1273, chronic inflammatory demyelinating polyneuropathy (cidp), demyelinating disorder, demyelinating polyneuropathy, cidp, covid 19 vaccine complication, covid-19 vaccine, moderna

## Abstract

Massive efforts are being made to develop coronavirus disease 2019 (COVID-19) vaccines at an unprecedented rate. The vaccinations' adverse impact profile, on the other hand, has not been well established. Neurological complications are increasingly reported as a result of these vaccines. One such complication identified is immune-mediated inflammatory polyneuropathy, which affects peripheral nerves and neurons. We report a case of chronic inflammatory demyelinating polyneuropathy (CIDP) post-mRNA-1273 (Moderna) COVID-19 vaccine. Recognizing this complication and distinguishing it from Guillain-Barré syndrome enables timely initiation of treatment. Additionally, our report highlights a possible link between vaccination and subsequent development of CIDP, but conclusive evidence of a causal relationship requires more extensive studies.

## Introduction

The World Health Organization (WHO) declared coronavirus disease 2019 (COVID-19) as a global pandemic in March 2020. COVID-19 viral infection is caused by the severe acute respiratory syndrome coronavirus 2 (SARS-CoV-2) [1]. COVID-19 vaccines are being developed at a rapid pace. However, the vaccine's side effect profile is still undetermined. Complications involving the nervous system are increasingly being reported due to the result of these vaccines. One such adverse event reported is immune-mediated inflammatory polyneuropathy. It is further classified as acute or chronic depending on the presentation and duration of symptoms.

Viruses and vaccines can induce autoimmune neuropathies via various mechanisms, including antigen mimicry, cytokine overexpression, and activation of self-reactive T-cell clones, resulting in aberrant major histocompatibility complex (MHC) class II expression [2]. The linkage of the swine flu vaccine with Guillain-Barré syndrome (GBS) in 1976 highlighted the possibility of inflammatory neuropathy related to vaccination [3]. Subsequently, research revealed that the collective GBS risk from influenza was considerably higher than that associated with influenza vaccinations [4].

Numerous vaccines have been associated with various neurological problems, including seizures with the pertussis vaccine, meningoencephalitis with the Japanese encephalitis vaccine, and giant cell arteritis with the influenza vaccine. Transverse myelitis, encephalitis, GBS, and optic neuritis have been documented following vaccination with human papillomavirus, rubella, yellow fever, measles, tetanus, rabies, and hepatitis A or B [5]. COVID-19 vaccines are not exempt from such adverse effects. Due to the severity of the COVID-19 pandemic, massive efforts were made to shorten the timing of vaccine development. Neurological problems associated with SARS-CoV-2 vaccinations are rare [6]. In their comprehensive analysis, Chen et al. described various neurological adverse effects associated with routinely available COVID-19 vaccinations [7,8]. Most of the neurological symptoms reported following vaccination are mild and transient, such as headache, dizziness, myalgia, and paresthesia. A few case reports have described more serious neurological symptoms such as GBS, transverse myelitis, and cranial nerve neuropathies [9-11]. Garg and Paliwal reviewed published studies on COVID-19 vaccines associated with neurological complications. The review detailed published reports of various neuroinflammatory, neurovascular, peripheral nerve, and neuromuscular complications [11]. Such severe neurological manifestations were also reported in the Vaccine Adverse Event Reporting System (VAERS) regarding all the COVID-19 vaccines [12,13]. Fernandes et al. documented a case series of four patients. Two of them developed new-onset seizures and transverse myelitis following immunization with the Pfizer-BioNTech vaccine. Other two developed meningitis-retention syndrome and GBS following vaccination with the ChAdOx1 nCoV-19 (AstraZeneca) vaccine [14].

Outside VAERS of the CDC, the reported data on COVID-19 vaccination-related chronic inflammatory demyelinating polyneuropathy (CIDP) are scarce. As of March 2022, there are 22 reported events in VAERS in the USA when searched under CIDP and Moderna vaccine. Out of 22 reported events, one event is documented by a healthcare provider confirming CIDP, and one event is documented by ER provider suggestive of CIDP without further diagnostic confirmation. Eight events were documented by patients or family members without citing confirmation of diagnosis through a healthcare provider or any mention of administration of relevant therapeutics, but event descriptions were consistent with neurological symptomatology in line with CIDP. Three events reported by patients or family members cited the confirmation of diagnosis through a healthcare provider. Two events reported by patients were suggestive of a relapse of CIDP rather than a new occurrence. Seven events reported by patients were not consistent with CIDP or any neurological symptomatology. Therefore, probably wrongly reported under CIDP. There is no reported published report on CIDP associated with the Moderna vaccine.

We present a case of an elderly female who developed lower extremities weakness gradually over five months. Clinical, pathognomonic, lab, and electromyography (EMG) findings were consistent with CIDP. Outside a few reports in VAERS, this is the first case of CIDP documented following immunization with the Moderna COVID-19 (mRNA-1273) vaccine.

## Case presentation

The patient was a 66-year-old female with a past medical history significant for type 2 diabetes mellitus with neuropathy, hypertension, and hyperlipidemia, who presented to the hospital with a five-month history of progressive lower extremity weakness and difficulty walking, and frequent falls. Her A1C was normal, and she was treated with basal insulin, short-acting insulin, and metformin. Eight months prior to admission, the patient had received Moderna COVID-19 two vaccine series. She experienced mild fever and malaise after the second dose, followed by residual fatigue lasting several weeks. Three months after that, she woke up with no feeling in both her legs and right arm weakness. She was admitted to the hospital for a stroke workup. She was hospitalized for evaluation of a transient ischemic attack. Brain imaging was unremarkable. The stroke workup was negative. She was discharged on aspirin and atorvastatin with a neurology follow-up. She also had a urinary tract infection identified, which was treated with appropriate antibiotics.

Her leg weakness kept on gradually worsening over the next two months when she also started having electric, shooting pain moving upwards from toes to hips and both legs. She also started noticing more bilateral arm weakness. Further, over the course of the next three months, she had a faster progression of her symptoms. She started using a wheelchair, as she could not get up from the couch. Her frequency of falls gradually increased to one to two daily falls on average. Symptoms were now associated with numbness and tingling in bilateral upper extremities, poor oral intake, and unintentional weight loss of 40 pounds over eight months. Bilateral leg weakness followed a waxing and waning pattern with an overall gradual decline in her strength. She tried as-needed ibuprofen for her extremity pain. She was evaluated by neurology service and was found to have hyporeflexia on the exam. She had unremarkable cervical, thoracic, and lumbar spine magnetic resonance imaging (MRI). No abnormal spinal cord signal, intrathecal enhancement, or thecal sac stenosis was seen. She had an unremarkable screening colonoscopy.

She was subsequently hospitalized for further evaluation and underwent extensive workup. Physical examination revealed decreased motor strength in all extremities involving proximal and distal muscles, more in the legs than arms. Decreased sensations of light touch were noted in all extremities. She had absent deep tendon reflexes in both lower extremities. Muscle tone and bulk were normal. Her complete blood count, comprehensive metabolic panel, erythrocyte sedimentation rate, C-reactive protein, creatine kinase, and thyroid-stimulating hormone were unremarkable. Serum protein electrophoresis showed a questionable protein band that was suspicious for being a monoclonal protein in the gamma globulin region. Serum immunofixation revealed IgG kappa monoclonal protein and a slightly abnormal IgG lambda monoclonal protein, which was not apparent in the less sensitive serum electrophoresis study. Urine protein electrophoresis showed predominantly glomerular proteinuria with an additional protein band that could potentially be a monoclonal protein. Urine immunofixation revealed IgG kappa monoclonal protein. Vitamin B1, folate, vitamin D, copper, and vitamin B12 levels were within the normal range. Lumbar puncture revealed clear CSF and albuminocytological dissociation as demonstrated in Table 1. CSF protein was 237 mg/dL, glucose was 56 mg/dL, and total nucleated cell (TNC) count was 2. CSF angiotensin-converting enzyme was normal. Serology testing, including antinuclear antibody (ANA), anti-double-stranded DNA, HIV, and West Nile virus, was negative. CSF polymerase chain reaction (PCR) testing for West Nile virus was negative. Coronavirus SARS-CoV-2 PCR testing was negative. MRI of the brain was unremarkable.

**Table 1:** Results of cerebrospinal fluid (CSF) analysis showing albuminocytological dissociation

CSF parameters	Patient values	Normal values
Glucose	56 mg/dL	40-70 mg/dL
Protein	237 mg/dL	15-45 mg/dL
WBC count	2 cells/cumm	0-5 cells/cumm
RBC count	1 cell/cumm	<1 cells/cumm
Xanthochromia	Absent	Absent
Angiotensin-converting enzyme	1.4 units/L	0.0-2.5 units/L

Nerve conduction studies demonstrated an absent sural sensory nerve response. The peroneal motor nerve response distal latencies were prolonged with significantly reduced amplitudes and significantly slowed proximal conduction velocities. The tibial motor nerve response distal latencies were significantly prolonged with reduced amplitudes. The F response was absent on the right and prolonged on the left. The nerve conduction results were consistent with prolonged distal latencies and decreased amplitudes in the bilateral peroneal and tibial motor nerve conductions, absent right sural sensory potential, and widespread active and chronic denervation in all muscles tested in both lower extremities. This pattern supported the diagnosis of CIDP with both axonal and demyelinating features. Concentric needle electromyography of the lower extremities demonstrated diffusely abnormal insertional activity with both fibrillations and positive sharp waves in all the tested muscles. The activated motor units of the lower extremities demonstrated increased motor unit complexities, increased amplitudes, and reduced recruitment patterns. In conclusion, electrodiagnostic findings were consistent with severe bilateral neuropathy with acute and chronic denervation changes consistent with CIDP. There were no findings to suggest myopathy or myositis (Tables 2-5).

**Table 2:** Sensory nerve conduction study ms: millisecond; NP: nerve potential; µV: microvolt; cm: centimeter; NR: no response.

Nerve/sites	Recording site	Peak Lat (ms)	NP Amp (µV)	Distance (cm)
Right sural (antidromic)				
Calf	Ankle	NR	NR	14
Reference		≤4.40	≥6.0	

**Table 3:** Motor nerve conduction study ms: millisecond; mV: millivolt; cm: centimeter; m/s: meters per second; EDB: extensor digitorum brevis; AH: abductor hallucis; R: right; L: left.

Nerve/sites	Muscle	Latency (ms)	Reference (ms)	Amplitude (mV)	Reference (mV)	Distance (cm)	Velocity (m/s)	Reference (m/s)
R peroneal - EDB								
Ankle	EDB	6.88	≤6.50	0.4	≥2.0	8		
Below fibular head	EDB	15.85		0.2	≥2.0			≥44
L peroneal - EDB								
Ankle	EDB	8.02	≤6.50	0.3	≥2.0	8		
Below fibular head	EDB	16.06		0.2	≥2.0	27	34	≥44
R tibial - AH								
Ankle	AH	10.71	≤6.50	0.2	≥4.0	8		
L tibial - AH								
Ankle	AH	11.85	≤6.50	0.2	≥4.0	8		

**Table 4:** F wave AH: abductor hallucis; ms: millisecond; R: right; L: left.

Nerve	M latency	F latency	Reference (ms)	F-M latency (ms)
R tibial - AH	0.0		≤57.5	
L tibial - AH	6.0	60.8	≤57.5	54.8

**Table 5:** Electromyography IA: insertional activity; PSW: positive sharp waves; PPP: polyphasic potentials; N: normal; R: right; L: left.

Muscle	IA	Fibrillation	PSW	Fasciculations	Amplitude	Duration	PPP	Pattern
R tibialis anterior	2+	2+	2+	None	1+	N	2+	Reduced
R gastrocnemius (medial head)	2+	2+	2+	None	1+	N	2+	Reduced
R peroneus longus	2+	2+	2+	None	1+	N	2+	Reduced
R vastus medialis	2+	2+	2+	None	1+	N	2+	Reduced
R iliopsoas	2+	2+	2+	None	1+	N	2+	Reduced
L tibialis anterior	2+	2+	2+	None	1+	N	2+	Reduced
L gastrocnemius (medial head)	2+	2+	2+	None	1+	N	2+	Reduced
L peroneus longus	2+	2+	2+	None	1+	N	2+	Reduced
L vastus medialis	2+	2+	2+	None	1+	N	2+	Reduced
L iliopsoas	2+	2+	2+	None	1+	N	2+	Reduced

She was treated with a 2 gm/kg dosing regimen of intravenous immunoglobulin (IVIG) therapy. She was discharged to a rehabilitation facility. At the three-month follow-up, significant improvement in her symptoms was noted. She was continued on IVIG treatment with a 0.6 gm/kg x two days every four weeks regimen.

## Discussion

Due to the significant prevalence of neurological diseases in the population and the vast number of vaccinated people, some of these conditions will manifest within the post-vaccination timeframe. As a result, establishing a causal link between vaccination and neurological disease will be a tough endeavor.

The current vaccines in use in the USA are Moderna (mRNA-1273), Pfizer-BioNTech (BNT162b2), and Johnson & Johnson's Janssen (Ad26.COV2.S). These vaccines are generally well-tolerated. Mild symptoms such as pain at the injection site, redness, fevers, headaches, or myalgias are common side effects. Numerous neurological adverse effects have been recorded following the administration of all current COVID-19 vaccines, most notably after Ad26.COV2.S and ChAdOx1 nCoV-19 (Oxford/AstraZeneca). While the occurrence of GBS is recognized in COVID-19 patients, several GBS cases have been associated with COVID-19 vaccines, particularly those containing adenoviral components. Neuropathies following vaccination are uncommon, and CIDP development during the post-vaccination interval is exceedingly rare, occurring in less than 2% of the individuals [15]. Moreover, CIDP occurrence remains poorly characterized [16]. Kim et al. published a case series of 13 GBS patients and variants following COVID-19 vaccination in South Korea [17]. Out of 13 patients, eight received AstraZeneca, and five received the Pfizer-BioNTech vaccine. While GBS is usually associated with adenovirus vaccines, it has been documented in relation to mRNA vaccines on multiple occasions [18-20]. Oo et al. reported inflammatory demyelinating polyneuropathy after receiving the ChAdOx1 nCoV-19 vaccine in four patients. Three patients had new-onset GBS, and one patient had a flare-up of GBS symptoms [21]. Bagella et al. described a case of GBS after ChAdOx1 nCoV-19 vaccination that progressed into CIDP [22]. Taga and Lauria recently published a review article in February 2022 about the peripheral nervous system and COVID-19 [23]. The authors conducted an extensive search of databases. Interestingly, they did not discover any report linking COVID-19 to a new onset CIDP. Few reports mentioned the exacerbation of CIDP symptoms with COVID-19 infection in known CIDP patients. Notably, because the GBS patients in the study were not tracked prospectively, it is unknown whether some of them had acute-onset CIDP, which has been described in up to 5-16% of patients in pre-COVID-19 investigations [24]. The authors were able to note a similar pattern of GBS incidence in post-COVID-19 vaccination patients. Also, they did not notice exacerbation of CIDP post-COVID-19 vaccination in existing CIDP patients. There is convincing evidence regarding acute inflammatory demyelinating polyneuropathy (AIDP) following vaccination with COVID-19 and COVID-19 vaccines (including Moderna). Abo-Zed and Pinevich reported a case of GBS that developed shortly after the Moderna COVID-19 vaccine, which subsequently evolved into CIDP [25]. Of note, this patient had a history of GBS/AIDP four years ago after receiving the influenza vaccine. Whereas our case did not have any history of AIDP/GBS or CIDP. Souza et al. reported a case series of four patients with CIDP after the ChAdOx1 nCoV-19 (AstraZeneca) vaccine [26]. While there have been a few studies examining the relationship between immunizations and chronic inflammatory neuropathies, most of them had limitations such as recall bias, retrospective design, and difficulty in precisely establishing the onset of disorders.

GBS and CIDP are immune-mediated illnesses in which the precise mechanism behind the pathophysiology of the immune response is unknown. GBS pathogenic episodes can generate antibodies against peripheral nerves and myelin sheath epitopes, leading to an autoimmune process. CIDP is often an idiopathic condition in which an immune response is directed against myelin components due to an autoimmune process involving primarily immunological mechanisms mediated by T cells. Hence, cellular immune mechanisms are a vital feature of CIDP. Infiltration of macrophages into nerves and perivascular inflammation on nerve biopsies imply that cell-mediated mechanisms are involved in demyelination. CIDP patients may have elevated T helper cells in the CSF [27]. Segmental demyelination and remyelination are characteristics of CIDP and repeatedly occur over time, resulting in onion bulb development via Schwann cell process growth.

The distinction between GBS and CIDP is important. GBS is the most prevalent cause of acute flaccid paralysis, characterized by autonomic dysfunction, sensory abnormalities, and varying degrees of weakness. Although the specific pathophysiology is unknown, this disorder is believed to result from an autoimmune response. GBS is often preceded by a respiratory or gastrointestinal infection. CIDP is a chronic disorder and the most common autoimmune polyneuropathy in adults. As per established criteria, a time period of more than eight weeks to recognize the greatest weakness in CIDP is used to differentiate it from AIDP, which reaches a nadir in four weeks or less. CIDP is characterized by a subacute to chronic onset that typically takes more than eight weeks to manifest paresthesias, loss of sensations, proximal and distal weakness, areflexia, asymmetric conduction velocity slowing with conduction-block like characteristics on electrodiagnostic testing, and association with albuminocytologic dissociation (elevated CSF protein without pleocytosis) [28]. Nerve pathology in CIDP shows segmental demyelination, onion bulb formation, perivascular inflammation, and axonal degeneration [28]. Patients often present with a relapsing and remitting course. Compared to AIDP, autonomic symptoms and back pain are less common in CIDP. In comparison to AIDP, the occurrence of respiratory failure leading to ventilator support or bulbar involvement is rare in CIDP.

CIDP is diagnosed based on specific clinical history and symptoms. CSF analysis, electrophysiological studies, and nerve biopsies are all important diagnostics, beneficial in excluding other possible diagnoses than in identifying CIDP. The absence of electrophysiological or histological evidence consistent with CIDP does not preclude the diagnosis. Similar caution should be exercised when interpreting demyelinating characteristics in nerve conduction studies or nerve biopsies performed on patients without clinical evidence of CIDP. CIDP has a variable prognosis like multiple sclerosis due to its heterogeneity. On average, 20-65% of patients experience a relapsing and remitting course, and the remainder experience a progressive course [29]. Most of the individuals with CIDP who do not have comorbid diseases respond to treatment, even more so when their CSF protein level is elevated.

The objective of this case report is to give essential information about the occurrence of new-onset CIDP, its clinical course, and diagnostic data that may be relevant in the management of comparable post-COVID-19 vaccination presentations. The progression of this patient's symptoms (both motor and sensory) was far more rapid than would be expected with diabetic polyneuropathy. Her symptoms were a bit too symmetric for diabetic amyotrophy. Around 90% of patients with CIDP have a high CSF protein level (more than 45 mg/dL) without CSF pleocytosis [30-32], which differentiates CIDP from multifocal motor neuropathy (MMN), which has a normal CSF protein level [33]. Albuminocytologic dissociation verifies the presence of immune-mediated polyneuropathy. Our diagnosis was further substantiated by nerve conduction studies and electromyography findings. The diagnosis of subacute GBS was considered in the differential. However, characteristics such as the lack of a distinct onset of symptoms and lack of autonomic symptoms went against the possibility of subacute GBS. Additionally, the patient showed symptoms three months after receiving the second dose of COVID-19 vaccination. The symptoms peaked sometime during the fifth month following their initiation. This goes against GBS, where symptoms nadir around eight weeks or less.

In our case, the relationship between the second dose of the mRNA-1273 (Moderna) vaccination and the onset of neuropathy is very evident, implying that the vaccine may have a triggering effect. This case report contributes to the limited body of evidence suggesting that immunization may contribute to the etiology of CIDP by providing one of the first detailed accounts of CIDP provoked by the Moderna vaccine. A close temporal link between vaccination, symptom onset, and a thorough diagnostic work-up to rule out other possible causes met the WHO criteria for identifying the causality of an adverse event following immunization on an individual basis. This led to the conclusion that this adverse event was most likely caused by the immunization.

## Conclusions

Our observations, combined with those of earlier case reports, indicate that COVID-19 vaccinations may be associated with inflammatory neuropathies. Awareness of this uncommon but possible adverse effect is critical for prompt diagnosis and treatment. The occurrence of CIDP described in this case coincided with the Moderna COVID-19 vaccine. More research is needed to determine whether our patient's clinical presentation was just a coincidence or if there is a cause-and-effect relationship. This is the first reported case of CIDP, which developed post-mRNA-1273 (Moderna) vaccination outside of very few reports in VAERS. At this point, the true link between this case and COVID-19 vaccination remains uncertain. Further work will be required in the future to ascertain a true association between COVID-19 vaccinations and CIDP rather than a mere coincidental finding. When comparing the incidence of CIDP following vaccination to the incidence of CIDP occurring spontaneously, large observational studies will be required to establish a causal relationship. As we learn more about the long-term consequences of COVID-19 vaccines, adding any such information to clinical databases and research is incredibly valuable.
